# Supplementary material for: A hitchhiker guide to manta rays: Patterns of association between Mobula alfredi, M. birostris, their symbionts, and other fishes in the Maldives
Source: PLoS One. 2021 Jul 14;16(7):e0253704. doi: 10.1371/journal.pone.0253704 (PMC8279400; doi:10.1371/journal.pone.0253704)
Supplement: S1 Table — Includes all combinations of explanatory variales for each hitchhiker species identified with Mobula alfredi and M. birostris. (PDF) [file pone.0253704.s001.pdf]

S1 Table. Summary of AICc relative goodness of fit metric values from the GLMM modelling procedure. Includes all combinations of explanatory variables for each hitchhiker species identified with *Mobula alfredi* and *M. birostris.*

***Mobula* spp.**	**Hitchhiker Species**	**Model**	**Class**	**df**	**logLik**	**AICc**	**ΔAICc**	**wAICc**
Mobula alfredi	*Gnathanodon speciosus*	Site.function + Maturity.Status	GLMM	6	-3090.35	6192.70	0.00	0.39
		Site.function + Monsoon + Maturity.Status	GLMM	7	-3089.83	6193.67	0.96	0.24
		Maturity.Status	GLMM	4	-3092.92	6193.84	1.14	0.22
		Monsoon + Maturity.Status	GLMM	5	-3092.84	6195.69	2.99	0.09
		Site.function + Gender + Maturity.Status	GLMM	10	-3089.80	6199.60	6.90	0.01
		Gender + Maturity.Status	GLMM	8	-3092.10	6200.21	7.51	0.01
		Site.function + Monsoon + Gender + Maturity.Status	GLMM	11	-3089.38	6200.77	8.07	0.01
		Site.function	GLMM	4	-3096.94	6201.89	9.18	0.00
		Gender	GLMM	6	-3094.99	6201.99	9.28	0.00
		Monsoon + Gender + Maturity.Status	GLMM	9	-3092.05	6202.10	9.40	0.00
		Site.function + Gender	GLMM	8	-3093.23	6202.47	9.77	0.00
		Monsoon	GLMM	3	-3098.46	6202.92	10.22	0.00
		Site.function + Monsoon	GLMM	5	-3096.49	6202.98	10.27	0.00
		Monsoon + Gender	GLMM	7	-3094.92	6203.83	11.13	0.00
		Site.function + Monsoon + Gender	GLMM	9	-3092.96	6203.92	11.22	0.00
		Site.function + Monsoon + Maturity.Status	GLM	6	-3148.79	6309.58	116.88	0.00
		Site.function + Maturity.Status	GLM	5	-3150.30	6310.60	117.90	0.00
		Site.function + Monsoon + Gender + Maturity.Status	GLM	10	-3148.25	6316.50	123.80	0.00
		Site.function + Gender + Maturity.Status	GLM	9	-3149.77	6317.55	124.85	0.00
		Site.function + Monsoon + Gender	GLM	8	-3158.71	6333.42	140.72	0.00
		Site.function + Gender	GLM	7	-3160.07	6334.14	141.44	0.00
		Site.function + Monsoon	GLM	4	-3163.77	6335.55	142.84	0.00
		Site.function	GLM	3	-3165.23	6336.46	143.76	0.00
		Maturity.Status	GLM	3	-3166.15	6338.30	145.60	0.00
		Monsoon + Maturity.Status	GLM	4	-3166.13	6340.26	147.56	0.00
		Gender + Maturity.Status	GLM	7	-3165.65	6345.30	152.60	0.00
		Monsoon + Gender + Maturity.Status	GLM	8	-3165.63	6347.26	154.56	0.00
		Gender	GLM	5	-3173.26	6356.53	163.82	0.00
		Monsoon + Gender	GLM	6	-3173.25	6358.49	165.79	0.00
		Monsoon	GLM	2	-3177.54	6359.08	166.37	0.00
		Maturity.Status + Monsoon + Pregnancy + Site.function	GLMM	11	-17639.08	35300.17	0.00	0.99
		Monsoon + Pregnancy + Site.function	GLMM	9	-17646.60	35311.20	11.02	0.00
		Maturity.Status + Pregnancy + Site.function	GLMM	10	-17653.06	35326.12	25.95	0.00
		Pregnancy + Site.function	GLMM	8	-17660.13	35336.26	36.09	0.00
		Monsoon + Site.function	GLMM	5	-17745.52	35501.04	200.87	0.00
		Maturity.Status + Monsoon + Site.function	GLMM	7	-17743.67	35501.35	201.17	0.00
		Site.function	GLMM	4	-17756.83	35521.67	221.50	0.00
		Maturity.Status + Site.function	GLMM	6	-17755.09	35522.19	222.02	0.00
		Maturity.Status + Monsoon + Pregnancy	GLMM	9	-17803.57	35625.14	324.97	0.00

Mobula alfredi	*Echeneis naucrates*	Monsoon + Pregnancy	GLMM	7	-17819.36	35652.71	352.54	0.00
		Maturity.Status + Pregnancy	GLMM	8	-17861.72	35739.44	439.26	0.00
		Pregnancy	GLMM	6	-17880.19	35772.39	472.22	0.00
		Maturity.Status + Monsoon	GLMM	5	-17919.76	35849.52	549.35	0.00
		Monsoon	GLMM	3	-17927.47	35860.94	560.77	0.00
		Maturity.Status	GLMM	4	-17976.52	35961.04	660.87	0.00
		Maturity.Status + Monsoon + Pregnancy + Site.function	GLM	10	-20446.42	40912.84	5612.67	0.00
		Monsoon + Pregnancy + Site.function	GLM	8	-20528.86	41073.72	5773.54	0.00
		Maturity.Status + Pregnancy + Site.function	GLM	9	-20603.91	41225.82	5925.65	0.00
		Pregnancy + Site.function	GLM	7	-20685.13	41384.26	6084.08	0.00
		Maturity.Status + Monsoon + Site.function	GLM	6	-20879.86	41771.73	6471.56	0.00
		Monsoon + Site.function	GLM	4	-20896.58	41801.16	6500.99	0.00
		Maturity.Status + Site.function	GLM	5	-21012.08	42034.17	6734.00	0.00
		Site.function	GLM	3	-21028.09	42062.19	6762.01	0.00
		Maturity.Status + Monsoon + Pregnancy	GLM	8	-21312.90	42641.80	7341.63	0.00
		Monsoon + Pregnancy	GLM	6	-21472.44	42956.89	7656.71	0.00
		Maturity.Status + Monsoon	GLM	4	-21812.49	43632.99	8332.82	0.00
		Monsoon	GLM	2	-21882.53	43769.06	8468.89	0.00
		Maturity.Status + Pregnancy	GLM	7	-21952.90	43919.81	8619.64	0.00
		Pregnancy	GLM	5	-22123.97	44257.93	8957.76	0.00
		Maturity.Status	GLM	3	-22404.74	44815.47	9515.30	0.00
Mobula alfredi	Juvenile *Echeneis naucrates*	Maturity.Status + Monsoon + Pregnancy + Site.function	GLMM	11	-3651.39	7324.79	0.00	1.00
		Maturity.Status + Monsoon + Site.function	GLMM	7	-3663.07	7340.14	15.35	0.00
		Maturity.Status + Pregnancy + Site.function	GLMM	10	-3662.33	7344.66	19.86	0.00
		Monsoon + Pregnancy + Site.function	GLMM	9	-3665.89	7349.79	24.99	0.00
		Monsoon + Site.function	GLMM	5	-3674.90	7359.80	35.01	0.00
		Maturity.Status + Site.function	GLMM	6	-3676.66	7365.31	40.52	0.00
		Pregnancy + Site.function	GLMM	8	-3678.36	7372.72	47.93	0.00
		Site.function	GLMM	4	-3687.66	7383.33	58.53	0.00
		Maturity.Status + Monsoon + Pregnancy	GLMM	9	-3691.79	7401.57	76.78	0.00
		Maturity.Status + Pregnancy	GLMM	8	-3694.69	7405.38	80.59	0.00
		Maturity.Status + Monsoon	GLMM	5	-3707.63	7425.25	100.46	0.00
		Maturity.Status	GLMM	4	-3711.07	7430.14	105.35	0.00
		Monsoon + Pregnancy	GLMM	7	-3712.92	7439.85	115.05	0.00
		Pregnancy	GLMM	6	-3715.41	7442.82	118.03	0.00
		Monsoon	GLMM	3	-3722.98	7451.97	127.18	0.00
		Maturity.Status + Monsoon + Pregnancy + Site.function	GLM	10	-3940.16	7900.32	575.52	0.00
		Maturity.Status + Monsoon + Site.function	GLM	6	-3967.35	7946.69	621.90	0.00
		Monsoon + Pregnancy + Site.function	GLM	8	-3975.81	7967.62	642.82	0.00
		Maturity.Status + Pregnancy + Site.function	GLM	9	-3977.89	7973.79	648.99	0.00
		Monsoon + Site.function	GLM	4	-3993.95	7995.90	671.11	0.00
		Maturity.Status + Site.function	GLM	5	-4010.15	8030.30	705.51	0.00
		Pregnancy + Site.function	GLM	7	-4018.32	8050.63	725.84	0.00
		Maturity.Status + Monsoon + Pregnancy	GLM	8	-4022.22	8060.44	735.64	0.00

		Maturity.Status + Pregnancy	GLM	7	-4031.10	8076.20	751.41	0.00
		Site.function	GLM	3	-4039.05	8084.10	759.31	0.00
		Maturity.Status + Monsoon	GLM	4	-4055.79	8119.59	794.80	0.00
		Maturity.Status	GLM	3	-4066.15	8138.30	813.50	0.00
		Monsoon + Pregnancy	GLM	6	-4076.06	8164.12	839.33	0.00
		Pregnancy	GLM	5	-4084.45	8178.89	854.10	0.00
		Monsoon	GLM	2	-4098.13	8200.25	875.46	0.00
Mobula alfredi	*Lutjanus bohar*	Site.function	GLMM	4	-908.49	1824.99	0.00	0.56
		Site.function + Monsoon	GLMM	5	-908.40	1826.79	1.80	0.23
		Site.function + Maturity.Status	GLMM	6	-908.16	1828.31	3.32	0.11
		Pregnancy + Site.function	GLMM	8	-906.99	1829.97	4.98	0.05
		Site.function + Monsoon + Maturity.Status	GLMM	7	-908.07	1830.13	5.14	0.04
		Pregnancy + Site.function + Monsoon	GLMM	9	-906.89	1831.78	6.79	0.02
		Pregnancy + Site.function + Monsoon + Maturity.Status	GLMM	11	-906.61	1835.22	10.23	0.00
		Pregnancy + Site.function + Maturity.Status	GLMM	10	-917.38	1854.76	29.77	0.00
		Monsoon	GLMM	3	-962.10	1930.20	105.21	0.00
		Monsoon + Maturity.Status	GLMM	5	-962.02	1934.05	109.06	0.00
		Pregnancy + Monsoon	GLMM	7	-960.61	1935.22	110.23	0.00
		Maturity.Status	GLMM	4	-965.07	1938.15	113.16	0.00
		Pregnancy + Monsoon + Maturity.Status	GLMM	9	-960.29	1938.58	113.59	0.00
		Pregnancy	GLMM	6	-963.48	1938.95	113.96	0.00
		Site.function	GLM	3	-967.97	1941.94	116.95	0.00
		Site.function + Maturity.Status	GLM	5	-966.30	1942.61	117.62	0.00
		Site.function + Monsoon	GLM	4	-967.32	1942.64	117.65	0.00
		Pregnancy + Maturity.Status	GLMM	8	-963.32	1942.65	117.66	0.00
		Site.function + Monsoon + Maturity.Status	GLM	6	-965.57	1943.13	118.14	0.00
		Pregnancy + Site.function	GLM	7	-966.34	1946.68	121.69	0.00
		Pregnancy + Site.function + Monsoon	GLM	8	-965.61	1947.21	122.22	0.00
		Pregnancy + Site.function + Maturity.Status	GLM	9	-964.97	1947.95	122.96	0.00
		Pregnancy + Site.function + Monsoon + Maturity.Status	GLM	10	-964.15	1948.31	123.32	0.00
		Monsoon	GLM	2	-1131.78	2267.55	442.56	0.00
		Monsoon + Maturity.Status	GLM	4	-1131.40	2270.81	445.82	0.00
		Pregnancy + Monsoon	GLM	6	-1130.96	2273.92	448.93	0.00
		Pregnancy + Monsoon + Maturity.Status	GLM	8	-1130.46	2276.93	451.94	0.00
		Maturity.Status	GLM	3	-1141.77	2289.54	464.55	0.00
		Pregnancy	GLM	5	-1141.34	2292.67	467.68	0.00
		Pregnancy + Maturity.Status	GLM	7	-1140.72	2295.45	470.46	0.00
Mobula birostris	*Remora remora*	Monsoon + Sex	GLMM	4	-483.20	978.51	0.00	0.30
		Maturity.Status + Monsoon + Sex	GLMM	6	-485.47	979.00	0.49	0.24
		Maturity.Status + Monsoon + Sex	GLM	5	-484.96	980.01	1.50	0.14
		Monsoon + Sex	GLM	3	-487.32	980.68	2.17	0.10
		Maturity.Status + Sex	GLMM	5	-485.54	981.16	2.65	0.08
		Sex	GLMM	3	-487.82	981.68	3.17	0.06
		Maturity.Status + Sex	GLM	4	-487.47	982.99	4.48	0.03
		Sex	GLM	2	-489.85	983.71	5.20	0.02
		Monsoon	GLMM	3	-489.81	985.66	7.15	0.01
		Monsoon	GLM	2	-491.64	987.31	8.79	0.00

		Maturity.Status + Monsoon	GLMM	5	-489.26	988.61	10.10	0.00
		Maturity.Status + Monsoon	GLM	4	-491.03	990.12	11.61	0.00
		Maturity.Status	GLMM	4	-494.67	997.40	18.89	0.00
		Maturity.Status	GLM	3	-496.77	999.57	21.06	0.00
