# Supplementary material for: A hitchhiker guide to manta rays: Patterns of association between Mobula alfredi, M. birostris, their symbionts, and other fishes in the Maldives
Source: PLoS One. 2021 Jul 14;16(7):e0253704. doi: 10.1371/journal.pone.0253704 (PMC8279400; doi:10.1371/journal.pone.0253704)
Supplement: Attachment — Submitted filename: Response to Reviewers.docx [file pone.0253704.s002.docx]

**Response to Reviewer’s Comments**

**Manuscript Number: PONE-D-21-04827**

Thank you for providing informative feedback. Please, find the comments included in your email below in black and our response in red.

1. Please amend your list of authors on the manuscript to ensure that each symbol is linked to an affiliation.

**Response:** The title page has now been amended, and each symbol has been matched to an affiliation.

2. We note that you have provided funding information that is not currently declared in your Funding Statement.

**Response:** We have not received any funding from the dive operator partners within the Maldives. This sentence has been adjusted to acknowledge their support, but not so it is interpreted as funding. The acknowledgements now read "We thank the Save Our Seas Foundation and the Manta Trust’s resort and dive operator partners in the Maldives for their overall support for the study". The funding statement can stay the same.

3. Your ethics statement should only appear in the Methods section of your manuscript.

**Response:** The ethics statement has been removed from the acknowledgements section to the methods.

4. We note that you have included the phrase “data not shown” in your manuscript (page 14, line 289). Unfortunately, this does not meet our data sharing requirements.

**Response:** This has been removed as the data is not a care part of the research being presented.

We are very grateful for the comments and suggestions provided by the editors. Please see below, in red, our detailed response to comments (in black). All line numbers refer to the manuscript file with tracked changes.

***Response to Reviewer #1 Comments***

Thank you for your positive and supportive feedback. Your comments are greatly appreciated and have allowed us to improve our manuscript.

Line 66-68: Consider rewriting. It’s not only megafauna but also deep-sea species, cryptic species etc.

**Response:** The sentence has been revised accordingly.

Line 69-71: However, our understanding of marine symbionts remains limited due to the logistical challenges associated with studying complex associations in mobile organisms over large spatial scales [21,24,25].

Lines 96-98: I don’t find this a logic statement and/or necessary. I suggest deleting this sentence and to simply continue with “Here, we …”

**Response:** Sentence removed as suggested.

Line 127: Adults/juveniles needs a reference.

**Response:** Reference added.

Line 147-150: Identified sharksucker remora (*Echeneis naucrates*) were further classified as either adults (>20 cm) or juveniles (≤20 cm) based on visual estimates against the host size [39], as well as differences between their colour and body patterns (Fig 2).

Line 143: (log10 + 1) Why is this here, what does this mean?

**Response:** The data were transformed (log10+1) due to the extreme difference between the high and low values i.e., the total number of sightings between *E. naucrates* in comparison to *N. doctor*). The transformation allows for better visualisation. We have added an explanation to the Fig 3. caption.

Line 243-244: A (*log*_10_+1)transformation was used for better visualisation of the data.

Lines 352-356: The interspecific relationship between echeneids and their hosts remains difficult to classify, is complex, and likely not a simplistic symbiotic relationship; see e.g. Brunnschweiler et al. 2020 “The costs of cohabiting: the case of sharksuckers and their hosts at shark provisioning sites”. I suggest the authors discuss their results a bit more in this context, i.e. how do their (interesting!) findings add to our knowledge/understanding of the costs and benefits of the association for both organisms. Another paper, Norman et al. 2021 “Three-way symbiotic relationships in whale sharks”, published just a few days ago, might also be of interest to the authors. Did they, e.g. observe *Remora remora* feeding on plankton like Norman et al. 2021 did for R. remora associated with feeding whale sharks in the Maldives?

**Response:** Thank you for highlighting the new publications. These have been revised and incorporated throughout the manuscript accordingly. The authors agree that the echeneid-host relationship is complex and varies between species and life stages. This has now been acknowledged more clearly in the manuscript. The authors have witnessed *E. naucrates* feeding at the same time as *M. alfredi* during night-time feeding events, as well as *R. albescens.* However, these are unnatural feeding events, so there must be a note of caution to any assumptions made. Certainly, *E. naucrates* and *R. remora* take advantage of being close to their host to forage. Solleliet-Ferriera *et al.* 2019 also identified *R. remora* preying jointly with sicklefin devilrays on baitfish. As the costs and benefits of the echeneid-host remain debated, further investigation into these aspects has also been highlighted as a potential direction for future studies.

Line 421-424: The relationship with their hosts is generally considered mutualistically symbiotic, as most remora species spend all their post-larval life in close association with their hosts, eating their ectoparasites in return for a range of benefits [15,39,40,58]. However, the degree of host specificity and the nature of the association has been shown to vary along the symbiotic continuum (mutualism, commensalism, and parasitism), and the importance of this host food source varies between remora species and at different life stages [1,25,38,39]. Recent investigations into the echeneid-host association also suggest that there may be significant costs incurred by the host, such as persistent damage and scarring from the adhesive disc by which the remoras attach themselves to their hosts, as well as deforming injuries when smaller-sized remoras force themselves through the gill slits and other body openings of their mobulid host [15,23,59].

Line 564-569: Previous examination of the diet data revealed that parasitic copepods comprise a crucial part of the *R. remora* diet, but that the importance decreases as the remora increases in size [25,38,39]. Despite this, the obligate nature of *R. remora*, like that of juvenile *E. naucrates* [58], further suggests that echeneid population dynamics are influenced by the distribution patterns of its host [22,58].

Line 594-596: Furthermore, the potential hosts costs and benefits of different echeneid densities remains relatively unexplored [59]. Research into these factors could also provide further insight into the patterns of association identified within the current study.

***Response to Reviewer #2 Comments***

Thank you for your comprehensive review and your constructive feedback which has helped us to improve the manuscript.

Abstract

Line 24. I suggest changing to “hitchhiker fish species” (or including ‘fish’ somewhere in this sentence) on initial mention here so it’s clear you’re not referring to parasites

**Response:** Revised as suggested.

Line 23-25: Manta rays are often sighted in association with an array of smaller hitchhiker fish species, which utilise their hosts as a sanctuary for shelter, protection, and the sustenance they provide.

Lines 30-31. I suggest including which statistical methods were used here eg. “using Generalized Linear Mixed Effects Models”.

**Response:** The statistical method has been added to the sentence.

Line 30-33: Here, we explore the spatial and temporal variation in hitchhiker presence with *M. alfredi* and *M. birostris* throughout the Maldives and investigate the factors which may influence association using generalised linear mixed effects models (GLMM).

Lines 37-40. Perhaps add a brief sentence outlining main findings for *M. birostris* here.

**Response:** A sentence has been added.

Line 42-43: *Remora remora* were most likely to be present with female *M. birostris,* and a mean number of 1.5 ± 0.5 *R. remora* were observed per *M. birostris***.**

Introduction

Lines 59-60. “…rather than the species themselves” I see what you’re saying here but without the species themselves there would be no interaction. I suggest re-wording to something like “rather than each species in isolation” or even “Such inter-specific interactions determine…” (here and in abstract)

**Response:** Sentence revised accordingly (in the abstract and introduction).

Line 25-27: Species interactions, rather than the species at the individual level, determine the ecological processes that drive community dynamics, support biodiversity and ecosystem health.

Line 63-65: These interactions, rather than the species at the individual level, determine the ecological processes that drive community dynamics, support biodiversity and ecosystem health [19].

Line 70. *M. birostris* has a circumglobal distribution, while *M. alfredi* has a semi-circumglobal distribution

**Response:** Sentence revised accordingly.

Line 72-73: *M. birostris* has a circumglobal distristribution, while *M. alfredi* has a semi-circumglobal distribution; both in tropical and subtropical waters [26-28].

Line 77. Do you mean “supports globally significant populations”? A single population of both species suggests they interbreed with one-another.

**Response:** Sentence revised accordingly.

Line 81-82: The Maldives archipelago supports globally significant populations of both species of manta ray [27].

Line 85. “and there only”, delete ‘there’?

**Response:** Word deleted.

Line 89-90: and only for a few months each year (March-April) during the transition from the NE to the SW Monsoon [27,35].

Line 88-91. I suggest swapping the order of these sentences. First explain that re-sighting rates are low, which, combined with seasonality of sightings, suggests a transient population which may be visiting these atolls as part of a ‘migration’. Then go on to say that where they are travelling from/to remains unknown.

**Response:** Sentence structure has been revised as suggested.

Line 91-95: These southern Maldives sites are in close proximity to deep-water oceanic habitat [27], sites where this species are most frequently encountered throughout their range [36,37]. Re-sighting rates of individuals remain extremely low, which, combined with the seasonality of sightings, suggests a large transient population which utilises habitat away from the reef systems of the Maldives. Where the Maldives *M. birostris* originate from, or travel to, remains unknown [35].

Line 92. It would be useful to define the term ‘hitchhiker’ in the context of this study here, upon first mention. This way the reader knows exactly what you mean.

**Response:** The term ‘hitchhiker’ has now been defined.

Line 101-106: Manta rays are often observed in association with hitchhiker fish species, such as the golden trevally (*Gnathanodon speciosus*) and members of the family Echeneidae (remoras), that closely follow (within 1 m) or attach themselves to their manta ray host [15,23]. It has been suggested that hitchhiking behaviour evolved as a means to gain protection from predation, enhance foraging opportunities, increase locomotor efficiency, and increase encounters with conspecifics [15,17,38–42].

Line 96-97. I suggest simplifying to “Considering the widespread effects of anthropogenic activities on marine habitats”

**Response:** This sentence has been removed as suggested by reviewer #1.

Line 99. I suggest changing “presence” to “associations” or “in the presence of hitchhikers with.”

**Response:** Changed to “in the presence of hitchhikers with”.

Line 112-113: Here, we explore the spatial and temporal variation in the presence of hitchhikers with *M. alfredi* and *M. birostris.*

Line 102. associations (change to plural)

**Response:** Changed to plural.

Line 116: highlighting how these associations are structured

Line 103. What is a generative process? Do you mean the drivers of the associations? Consider re-wording

**Response:** Yes, the sentence has been revised accordingly.

Line 114-117: This study aims to improve our ecological understanding of interactions between manta rays and their hitchhikers by highlighting how these association are structured, and what the drivers of the associations might be [11].

Materials and Methods
Line 111-112. This is correct, but the basis for compiling photo-ID catalogues is that individuals can be distinguished from one-another, not just from an existing catalogue, as new individuals are added over time. I suggest re-wording here. Also include the term “photo-identification” in full here as it is the first mention, with the abbreviation in parentheses.

**Response:** The sentence has been revised accordingly.

Line 125-127: The unique ventral body pigmentation of each manta ray enables individuals to be distinguished from one-another using a photo-identification (photo-ID) catalogue of the ventral surface of the rays [26,27,46].

Line 119-120. Was species identification/sex/maturity status determined on the dive or from photos? Please include how you determined sex (presence/absence of claspers) and maturity status for each sex, or cite a study which includes more detail on this.

**Response:** The observer, when possible, recorded this information and added it to the sighting record during the dive/snorkel. Thereafter, two-step verification and further details were added through assessment of the image/s by trained research staff using the methods described in Stevens 2016 and Peel *et al.* 2018. The paragraph has been revised accordingly and further references added.

Line 133-139: Where possible each manta ray was identified to species [47], and the sex and maturity status (adult, subadult, juvenile) of each was recorded during the dive/snorkel. For each sighting, the pregnancy status (an estimate of trimester) and primary behavioural activity (cleaning, feeding, courtship, cruising, or breaching) [27,48] were also recorded. Thereafter, two-step verification and further sighting details were determined through assessment of the image/s by trained staff using the methods described in Stevens [27], and Peel *et al.* [49].

Line 122. What determined whether a fish was a hitchhiker? For example, compared to a cleaner fish or just another fish in the vicinity of the manta ray, it would be useful to outline this here.

**Response:** All Manta Trust research staff were trained to recognise the biological and ecological differences between cleaner fish (family Labridae) and other teleost species. For example, cleaner fish have conspicuous colour and pattern combinations that help distinguish them from other co-occurring species. The manta ray client seeks out cleaner species, the cleaner fish does not follow the manta ray away from the cleaning station, and the association is brief (approximately 5 minutes) (Murie *et al.* 2020). Whereas hitchhikers were classified as smaller-bodied species that closely follow (within 1 m) or attach themselves (Echeneids) to their manta ray host i.e., they will follow their host away from the cleaning station. Many of the species identified within the study have also been recorded to ‘hitchhike’ other large-bodied marine species i.e., species of the family Carangidae, pilot fish, cobia, and species of the well-known hitchhiking Echeneidae family (Fig 2). The term ‘hitchhiker’ has now been defined in the introduction, as suggested by a previous comment. A sentence has been added on how hitchhiker species were distinguished from cleaner fish.

Line 145-147: The conspicuous colour, patterns and behaviour of co-occurring cleaner fish (Labridae) enabled them to be clearly distinguished from the hitchhiker species [51].

Line 122-123. The presence/number/species of hitchhikers was recorded, was this done during the dive? This can be tricky when there are a lot of mantas in the water at the same time (>15), in these cases were hitchhiker number and species determined by the photos? Please clarify.

**Response:** When possible, the presence of hitchhiker species was recorded by the trained observer during the dive (especially for *M. birostris*, which often have remoras located dorsally on the manta rays as well as ventrally). However, the majority of hitchhikers were identified and counted using the images. Even if there were lots of manta rays present, it was easy to determine the host because the hitchhikers remain close to their host.

Line 143-145: The presence, number, and species of hitchhikers associated with each manta sighting were recorded during the dive/snorkel and verified by visual analysis of all photo-ID images (Fig 2), using FishBase to determine species identification [50].

Line 126. Please clarify whether the photo-ID images were ventral images, dorsal images or both. If only ventral, how did you assess the number of hitchhikers on the dorsal surface (as some species tend to move around the body of the host)? Or did you only include individuals that had both dorsal and ventral images?

**Response:** Photo-IDs are primarily ventral, but for each sighting, on average, there are 2.3 images per individual, allowing for greater hitchhiker capture. In the authors experience, the main species where not having dorsal images is a problem is for *R. remora* on *M. birostris*. In these sightings, the observers did try to note during the surveys how many remoras can be seen, and to take dorsal as well as ventral images of this species (as well as video).  We do acknowledge this as a potential limitation in the manuscript’s discussion. It is also acknowledged as a potential limitation for the golden trevally, which often locate themselves around the head of the manta ray, exhibiting ‘piloting behaviour’. Please see response to previous comments.

Line 126. If the count from the dive did not match the count from the photo, how was this resolved?

**Response:** If a specific observation record of the count was taken, this was used. If there were multiple images of an individual manta sighting which showed differing hitchhiker individuals (e.g., one image showed 4 remoras, another 5), the greatest confirmed visible number of hitchhikers was used.

Line 128. How was size measured? Was it estimated? If so, was this based on comparison with other objects of known size (perhaps the manta ray). Was there a way to resolve discrepancy between different observers and size estimates? As this can be variable among different people, particularly when estimating measurements underwater.

**Response:** This was a visual estimate. Yes, the manta ray was used as a comparison to estimate the size of the remoras. Juvenile *E. naucrates* are also a different colour to the adults, which greatly aided observers in categorizing age class. Adult remoras are typically very light grey in colour with a faint black stripe running through the eye; the stripe fading out along the midline of the body towards the tail. Juvenile *E. naucrates* are generally darker grey, with much more distinct separation between the body background colour and black midline stripe which runs along the entire length of the body. In juveniles, this central black line is also bordered with white (Fig 2). A sentence has been added to the manuscript.

Line 147-150: Identified sharksucker remora (*Echeneis naucrates*) were further classified as either adults (>20 cm) or juveniles (≤20 cm) based on visual estimates against the host size [39], as well as differences between their colour and body patterns (Fig 2).

Line 128-129. Was the entire photo/record of the individual manta ray removed from analysis, or just the unidentifiable hitchhiker?

**Response:** Only the unidentifiable hitchhiker species were not used in the analysis, the sighting of the individual manta ray was still included.

Figure 2. If these were the images used for identification, how were the images themselves identified? I suggest including a citation for the reference material used (i.e. a fish ID book).

**Response:** The images themselves were identified using the scientific database Fishbase ([www.fishbase.org](http://www.fishbase.org)). The manuscript has been revised accordingly.

Line 143-145: The presence, number, and species of hitchhikers associated with each manta sighting were recorded during the dive/snorkel and verified by visual analysis of all photo-ID images (Fig 2), using FishBase to determine species identification [50].

Line 143. Why were the data transformed (log10 +1)? Please outline briefly here.

**Response:** The data were transformed (log10 +1) due to the extreme difference between the high and low values, i.e.,the total number of sightings between *E. naucrates* in comparison to *N. doctor*). The transformation allows for better visualisation. We have added an explanation to the Fig 3. caption.

Line 243-244: A (*log*_10_+1) transformation was used for better visualisation of the data.

Line 145. ‘mean daily number of hitchhikers’ - is this all species combined? Or separated by species?

**Response:** Separated by species. The sentence has been revised accordingly.

Line 165-169: To investigate variations in the total number of the most frequently observed hitchhiker species present with *M. alfredi*, the difference in the daily mean number of each hitchhiker species (number of sightings / number of hitchhiker species observed) was compared among manta ray sex and pregnancy stage

Line 146. Here and elsewhere (line 219), I suggest changing ‘manta sexual group’. You could say ‘compared among sex and pregnancy stage’ or similar.

**Response:** Changed to ‘manta ray sex’.

Line 168-169: was compared among manta ray sex and pregnancy stage

Line 147. Add ‘season’ after Monsoon.

**Response:** The wording has been revised.

Line 171: season (NE or SW Monsoon)

Line 153. Please add a citation for the R package here.

**Response:** Citation added.

Line 176: and ‘oneway’ function of the ‘userfriendlysceince’ R package, respectively [52].

Line 155. Which were the most frequently observed species? Include them here.

**Response:** The sentence has been revised accordingly.

Line 178-179: Spatial variation in the presence of the most frequently observed hitchhiker species with *M. alfredi* (adult and juvenile *E. naucrates*) were investigated

Line 158. Change to ‘sightings’, plural.

**Response:** Changed to ‘sightings’.

Line 181-182: (total number of sightings where the hitchhikers were observed / total number of sightings at the site) in ArcGIS 10.7.

Line 159-160. How many sites were removed, and which sites?

**Response:** 213 sites had sightings less than or equal to 9. This has been added to the manuscript, but the names are not included as there are too many.

Line 182-183: Any sites with a total of nine or fewer sightings (213 sites) were excluded to reduce the bias a low number of sightings may have on analysis.

Line 168. How did you assess whether your variables fit the normality assumptions for a GLMM? Did you test for correlations between predictors? Please include this here.

**Response:** The DHARMa R package was used to check the model residuals were normally distributed. We have added this to the manuscript. Correlations between variables were not considered because the data is categorical, but models were tested for multicollinearity using the variance inflation factor (VIF). We have now included this in the manuscript.

Line 205-206: To compare the relative goodness-of-fit, GLMM models without random effects (GLM) were tested.

Line 212-213: The variance inflation factor (VIF) was used to test models for multicollinearity; the maximum VIF was <1.5.

Line 213-216: Model performance was assessed using corrected Akaike information criterion (AICc) test statistic [53] using the ‘MuMin’ R package [54], and the DHARMa R package [55] was used to check the model residuals were normally distributed.

Line 170-172. Is there a reason why estimated/measured disc width wasn’t assessed? Perhaps surface area may influence the number/size/species of hitchhikers.

**Response:** The data available only included rough size estimates for the majority of manta ray sightings. Therefore, the resolution of data was just not available for enough of the database to be statistically usable. The Manta Trust is now collecting further disc width data (using stereo-video photogrammetry) for future directed studies. The manuscript has been revised accordingly and manta ray size has been highlighted as an opportunity for future research within the discussion.

Line 590-594: Due to logistical constraints, accurate size data on the sighted manta rays during this study was rarely collected. Therefore, variation in manta ray disc width (DW) and hitchhiker presence was not investigated here. However, future directed studies could investigate DW (i.e., the sexual dimorphism between male and female manta rays) as a potential further pattern of association.

Line 173. Outline which species were the most frequently observed with *M. birostris.*

**Response:** Added to sentence as suggested.

Line 199-201: The same model was used for *R. remora* (the most frequently observed hitchhiker species with *M. birostris*), but without site function, and sex was classified only as male or female as pregnancies were only observed during four sightings.

Line 177. Do you mean pseudoreplication?

**Response:** Yes, each model included a random intercept to account for the true structure of randomness (the same individual manta ray being repeatedly sighted) and to eliminate pseudoreplication. A reference for the paper ‘Remedies for pseudoreplication’ has been added.

Line 203-205: Each model contained the manta-ID as a random intercept to account for any the temporal autocorrelation arising from individual rays being repeatedly observed [52].

Line 178. Is there a reason why the results of this goodness-of-fit comparison are not reported?

**Response:** The incorrect table was uploaded during submission. Thank you for bringing this to our attention. The correct table has now been uploaded.

Line 182-183. This sentence can be moved to the results section.

**Response:** The sentence has been removed as it was already included in the results.

Results
Line 196-200 and 254-255. Given the large numbers of sightings and individuals, percentages would be useful to include here.

**Response:** The manuscript has been revised accordingly with further percentage values added throughout.

Line 227-232: A total of 4901 *M. alfredi* were individually identified [male = 2442 (50%), female = 2459 (50%)] during a total of 72912 sightings, of which 44071 (60%) were of females [adult = 25700 (58%), juvenile = 18371 (42%)] and 28841 (40%) were males [adult = 25968 (90%), subadult = 1443 (5%), juvenile = 1430 (5%)]. All sightings occurred across 353 sites, of which 95 (27%) were cleaning stations [sightings = 24034 (33%)], 53 (15%) were cruising areas [sightings = 129 (0%)], and 205 (58%) feeding areas [sightings = 48749 (67%)].

Line 291-294: There was a total of 72361 sightings across 149 sites which had ten or more manta ray sightings; 23926 (33%) occurred at cleaning stations where *E. naucrates* were present 4474 (19%) of observations, and 48435 (67%) occurred at feeding areas where *E. naucrates* were present during 2641 (5%) of observations.

Line 203. Is this 10% of total sightings? Or sightings with hitchhikers? Please clarify.

**Response:** 10% of the total sightings. The sentence has been revised.

Line 234-236: The most frequently observed hitchhiker species with *M. alfredi* was *E. naucrates*. Adult *E.* *naucrates* were observed with *M. alfredi* during 7189 (10%) of the total sightings, and juveniles during 756 (1%) sightings.

Line 218, 237 and 335. What is meant by daily mean number? The total number of E. naucrates seen in a single day? Or is this by sighting/individual? Please clarify.

**Response:** Sighting/individual. The manuscript has been revised accordingly. Please also see response to previous comment.

Line 165-169: To investigate variations in the total number of the most frequently observed hitchhiker species present with *M. alfredi*, the difference in the daily mean number of each hitchhiker species (number of sightings / number of hitchhiker species observed) was compared among manta ray sex and pregnancy stage

Line 217 and line 236. Consider re-wording the opening sentences of these paragraphs. “The number of E. naucrates recorded ranged from 1-24 individuals”, or similar.

**Response:** Opening sentences have been re-worded as suggested.

Line 250-251: When present, the number of adult *E. naucrates* associated with *M. alfredi* ranged between one and twenty-four individuals per sighting.

Line 271-272: When present, the number of juvenile *E. naucrates* associated with *M. alfredi* ranged between one and four individuals per sighting.

Line 257: “The highest percentage of E. naucrates presence by atoll” Is this based on the total or mean per individual manta?

**Response:** This is based on the total, not the mean per individual manta ray.

Line 261-262. “*Echeneis naucrates* were not observed with *M. alfredi* during any sightings which occurred at 25 sites” Please re-word this sentence, do you mean that there were 25 sites at which *E. naucrates* were not observed?

**Response:** Yes, the sentence has been revised accordingly.

Line 298-300: *Echeneis naucrates* were not observed with *M. alfredi* at 25 sites, 17 of which are feeding areas (total sightings = 1512) and 8 cleaning stations (total sightings = 266).

Line 276-277. This belongs in methods section.

**Response:** Sentence moved to the methods section.

Line 197-198: Due to the low number of recorded associations between *M. alfredi* and most of the hitchhiker species, only those with sufficient data were included in the GLMM analysis.

Line 279-280. Reference Table S1 here. With regard to Table S1, it is useful to report delta AICc in model selection tables and I would advise doing so here.

The second highest ranking model has delta AICc of <2. According to Burnham and Anderson (2004), when the difference in AIC between two models (delta AICc) is < 2, then there is substantial support for the two models having approximately equal weight in the data, and this rule-of-thumb is commonly adopted in the model selection process. Please explain in the methods section how you treated models within delta AICc of <2.

Burnham KP, Anderson DR (2004) Multimodel inference: understanding AIC and BIC in model selection. Sociol Method Res 33:261-304

**Response:** As per previous comments, the incorrect S1 Table was uploaded by mistake during our initial submission. The correct table has now been submitted, which includes both delta and weights. Three species had models with delta AICc<2. We have now considered these models in our interpretation of the highest-ranking model. As such, the following changes have been made to the manuscript.

Line 316-317: The highest ranking GLMM (ΔAICc = 0) for *E. naucrates* contained all four predictors (site function, sex, maturity, and season) (S1 Table).

Line 218-219: Any models with ΔAICc <2 were considered in interpretation of the highest ranking model [55].

Line 327: There were no models with ΔAICc <2 (S1 Table).

Line 337: There were no models with ΔAICc <2 (S1 Table).

Line 343-345: There was one model with ΔAICc <2 (S1 Table), which was the same as the highest-ranking model with the addition of monsoon, but this predictor was non-significant (*p*>0.05).

Line 353-358: There were two models with ΔAICc <2 (S1 Table). One of these models contained the same predictors as the highest-ranking model with the addition of season, but this predictor was non-significant (p>0.05). The other model contained only the predictor maturity status, which suggested *G. speciosus* were more likely to be present on juvenile *M. alfredi* (OR = 1.5), which were 55% more likely to have this hitchhiker species than adults (reference category).

Line 538-539: The models also suggest that maturity status might be more influential than site function as one model with AICc<2 contained only the predictor maturity status.

Line 306. According to Table S1 the highest-ranking model (AICc = 6192.70) contains site function and maturity status as predictors, is the text correct or the table?

Response: The incorrect table was uploaded during the initial submission; the correct table has now been submitted. The AICc value should be Δ AICc, and the sentence should have said that there were two predictor variables. The manuscript has been changed accordingly.

Line 347-348: The highest ranking GLMM (ΔAICc = 0) for *G. speciosus* contained two predictors (site function and maturity status).

Line 275-311. The findings for the GLMM are the same as those for the Welch’s ANOVA and post-hoc tests for M. alfredi. Please explain why it is necessary to use both analyses.

**Response:** The Welch’s ANOVA was applied to the daily mean number of hitchhiker species present whereas the GLMM was performed using all sightings. By incorporating both these methods we feel it provides additional support for the results of each and further aides interpretation.

Line 344. See my comment above about delta AICc of <2 in Table S1.

**Response:** Please, see our response to the above comment.

Discussion
I suggest starting the discussion with a paragraph that clearly summarises your main findings. This will guide the structure of the rest of the discussion to follow.

**Response:** A paragraph has been added to the discussion.

Line 403-408: A variation in the species of hitchhiker associated with *M. alfredi* and *M. birostris* was identified, with *E naucrates* and *Remora remora* being the most common, respectively. Spatiotemporal variation in the presence of manta rays was identified as a driver for the occurrence of ephemeral hitchhiker associations, and for the first time, associations between *M. alfredi* and *M. birostris* with hitchhiker species other than those belonging to the family Echeneidae are described.

Line 378. *E. naucrates* were more likely to be associated with females. Have you considered the influence of sexual dimorphism? i.e., females are typically larger in size than males.

It would be interesting to assess whether there was any relationship between body size (disc width; if these data are available) and hitchhiker presence/abundance? Given that adult *E. naucrates* were more likely to be associated with adult *M. alfredi* (and the same with juvenile hosts and hitchhikers), is there a chance this is related to surface area? i.e. Larger surface area = more protection for larger hitchhikers.

This theory could also be applied to smaller hitchhiker species being more commonly associated with the smaller of the manta ray species (*M. alfredi/E. naucrates*) and larger hitchhikers with the larger of the manta species (*M. birostris/ R. remora*).

**Response:** *M. alfredi* are sexually dimorphic with females approximately up to 16% larger than males (Deakos 2010). Given that many individual *M. alfredi*, when sighted, have zero *E. naucrates*, it seems unlikely the remoras are seeking out the larger-bodied females to associate with, especially as these associations are ephemeral. As suggested within the study, the associations identified are likely the result of the ecology and habitat use variations/similarities between the species.

The data available only included rough size estimates for most of the individuals/sightings. Therefore, the resolution of data was just not available for enough of the database to be statistically usable. However, the Manta Trust is now collecting more size (disc width) data for future directed studies. The manuscript has been revised accordingly and manta ray size has been highlighted as an opportunity for future research within the discussion.

Please also see response to previous comment.

Line 590-596: Due to logistical constraints, accurate size data on the sighted manta rays during this study was rarely collected. Therefore, variation in manta ray disc width (DW) and hitchhiker presence was not investigated here. However, future directed studies could investigate DW (i.e., the sexual dimorphism between male and female manta rays) as a potential further pattern of association. Furthermore, the potential hosts costs and benefits of different echeneid densities remains relatively unexplored [59]. Research into these factors could also provide further insight into the patterns of association identified within the current study.

Line 383. Have you considered the nature of manta ray behaviour at different sites? For example, cleaning is characterised by slow movements and hovering, while feeding is typically faster movements, more turns etc. Could this make it harder for the hitchhikers to catch up/stay attached to their host?

**Response:** The adhesive disk of echeneids is capable of remaining attached to the surface of their host under high pressure conditions, where drag forces create pressure strain (Flammang *et al.* 2020). Recent studies have identified that remoras are able to sense when they have made contact with a host, and when to initiate rapid attachment to ensure long-term adhesion (Cohen *et al.* 2020). Although there is a difference in manta ray swimming speed between cleaning and feeding behaviours, it is minimal, as even when feeding, manta rays are still relatively slow swimmers, with turning manoeuvrability similar to that of swimmers with rigid bodies (Fish *et al.* 2018). The majority of the other hitchhiker species identified (i.e., trevallies, red snapper, cobia) are fast-swimming predatory fish. Therefore, it is unlikely that the movement of the manta ray creates challenges for the remora to stay attached to their host.

Line 417. How is predation risk reduced at cleaning stations?

**Response:** The majority of cleaning stations in the Maldives archipelago are situated on shallow reefs (<20m) (Stevens 2016; Harris *et al.* 2020). Stevens 2016, proposed that cleaning stations may act as a potential refuge from predators such as pelagic sharks and cetaceans because manta rays are more vulnerable to attacks from below in open and deeper water. When on these shallow reefs, the manta rays also stay close to the seabed. Similarly, predator avoidance has also been suggested to explain the habitat use of shallow lagoons by juvenile manta rays (McCauley *et al.* 2104; Stevens 2016; Germanov *et al.* 2019).

Line 418-435. This paragraph jumps between a number of themes/hypotheses and it distracts from the major point. Please re-word, and potentially separate the themes, for clarity.

**Response:** The themes within this paragraph are all linked to one main hypothesis. Anderson *et al.* 2011, suggested that there may be an increase in primary productivity during the NE Monsoon that occurs at a much deeper depth. Manta rays are physiologically able to dive at such depths due to a counter-current heat-exchange mechanism. However, to do so manta rays would have to bask in warmer shallow water to recover from the metabolic costs incurred from such deep foraging bouts. This behavioural thermoregulation has been used to explain why other elasmobranch species return to shallow habitats after deep dives. The paragraph structure has been revised accordingly.

Line 487-505: However, greater abundances of large skipjack (*Katsuwonus pelamis*) and yellowfin tuna (*Thunnus albacares*) have been observed in the Maldives in the less productive NE Monsoon [33]. This led Anderson et al. [33] to suggest that there may be an increase in primary productivity at this time associated with a deep chlorophyll maximum (DCM) that is not visible to the satellite technology (SeaWiFS) which records the high SW Monsoon productivity. Manta rays are poikilothermic, with an optimal thermal temperature of 20 – 26 ̊C, but can endure colder temperatures for short periods due to a counter-current heat-exchange mechanisms [63,74]. This physiological adaption enables manta rays to forage on zooplankton blooms within the deep-scattering layer, and down to depths of over 672 metres and temperatures of 7.6 ̊C [63]. If Anderson et al. [33] hypothesis is correct, basking in warmer shallow waters during the NE Monsoon, at sites like cleaning stations, prior to and post deep forays may enable manta rays to physiologically prepare for, and recover from, the large metabolic costs incurred from such deep foraging bouts [63,75]. This behavioural thermoregulation has also been used to explain why the spinetail devil ray (*Mobula mobular*) [76], the whale shark (*Rhincodon typus*) [75], and tuna species return to shallow habitats after deep dives [77]. To support this hypothesis, more data on the diving behaviour and habitat use of manta ray in the Maldives is required, utilising camera-mounted and satellite tracking technologies, along with chlorophyll-α measurements, to address this question.

Line 475 & 516-519. The fact that sightings images were primarily focused on the ventral surface of rays should be made clearer in the results section. See my comments above.

**Response:** This has been revised and made clearer in the methods section when discussing the photo-ID methodology. Please see previous comments.

Line 491. What do you mean by population structure here? Demographic structure i.e. the distribution of adults v juveniles? Please clarify.

**Response:** This sentence links to the discussion regarding *E. naucrates* on line 466. Bachman *et al.* 2018, proposed that hitchhiker behaviour restricts mate selection, reproduction, and location. Thus, echeneid population dynamics are influence by that of their host. There is limited literature on *R. remora* ecology and population dynamics. Therefore, the current study draws upon the suggestions of Bachman *et al.* 2018 for *E. naucrates* to suggest that the obligate nature of the *R. remora* provides evidence that the population dynamics of this species is also influenced by that of its host. Sentence reworded accordingly.

Line 566-569: Despite this, the obligate nature of *R. remora*, like that of juvenile *E. naucrates* [58], further suggests that echeneid population dynamics are population structure is also influenced by the distribution patterns of its host [22,58].

Line 499. Avoid referring to tables and figures in the discussion section.

**Response:** Reference to tables and figures have been removed from the discussion accordingly.
